# Environmental Challenges and Physiological Solutions: Comparative Energetic Daily Rhythms of Field Mice Populations from Different Ecosystems

**DOI:** 10.1371/journal.pone.0051247

**Published:** 2012-12-14

**Authors:** Michael Scantlebury, Abraham Haim

**Affiliations:** 1 Department of Biology, University of Haifa Oranim campus, Tivon, Israel; 2 School of Biological Sciences, Queen’s University Belfast, Belfast, United Kingdom; 3 The Israeli Centre for Interdisciplinary Research in Chronobiology, University of Haifa, Mount Carmel, Israel; University of Sydney, Australia

## Abstract

Daily and seasonal variations in physiological characteristics of mammals can be considered adaptations to temporal habitat variables. Across different ecosystems, physiological adjustments are expected to be sensitive to different environmental signals such as changes in photoperiod, temperature or water and food availability; the relative importance of a particular signal being dependent on the ecosystem in question. Energy intake, oxygen consumption (VO_2_) and body temperature (T_b_) daily rhythms were compared between two populations of the broad-toothed field mouse *Apodemus mystacinus*, one from a Mediterranean and another from a sub-Alpine ecosystem. Mice were acclimated to short-day (SD) ‘winter’ and long-day (LD) ‘summer’ photoperiods under different levels of salinity simulating osmotic challenges. Mediterranean mice had higher VO_2_ values than sub-Alpine mice. In addition, mice exposed to short days had higher VO_2_ values when given water with a high salinity compared with mice exposed to long days. By comparison, across both populations, increasing salinity resulted in a decreased T_b_ in SD- but not in LD-mice. Thus, SD-mice may conserve energy by decreasing T_b_ during (‘winter’) conditions which are expected to be cool, whereas LD-mice might do the opposite and maintain a higher T_b_ during (‘summer’) conditions which are expected to be warm. LD-mice behaved to reduce energy expenditure, which might be considered a useful trait during ‘summer’ conditions. Overall, increasing salinity was a clear signal for Mediterranean-mice with resultant effects on VO_2_ and T_b_ daily rhythms but had less of an effect on sub-Alpine mice, which were more responsive to changes in photoperiod. Results provide an insight into how different populations respond physiologically to various environmental challenges.

## Introduction

Ecological physiology aims to understand how organisms function in and respond to their natural environment [1]–[3]. In many cases, environmental stresses are not constant over time but take place on a cyclical/seasonal basis (e.g. daily or annually). Animals subsequently display variations in their behavioural and physiological traits, such as activity patterns and body temperature (T_b_) or oxygen consumption (VO_2_) daily rhythms; the latter reflecting changes in metabolic processes [4]–[6]. Such endogenous rhythms allow animals to inhabit different environments and can normally be entrained by a ‘Zeitgeber’, such as photoperiod [7]. In ecosystems that vary temporally, mammals often display daily and seasonal variations in their thermoregulatory system, which are considered necessary adaptations to their habitats [8], [9], [10]. Indeed, comparing physiological variables of closely related individuals from different habitats or ecosystems using chronobiological tools makes it possible to examine the potential outcome of natural selection for physiological characteristics [11]–[13]. Environmental adjustments of terrestrial animals to different habitats and different seasons may be achieved by seasonal acclimatization of the digestive, thermoregulatory and osmoregulatory systems [14]–[19]. Small mammals inhabiting challenging ecosystems with reduced water and food resources have lower energy demands and an increased digestive efficiency compared with those inhabiting mesic and more productive environments [20]–[22]. By comparison, those that inhabit seasonally cold environments regularly have increased metabolic capacities and energy requirements for thermoregulation [18], [23]–[25].

The Levant sub-region is one of extreme biogeographical heterogeneity; large seasonal variations in ambient temperature, water and food occur in a relatively restricted area [26]. Low-lying hills, such as the Mount Carmel ridge, are characterised by short, wet winters and long, hot and dry summers (8–9 months). By comparison, high mountainous areas such as Mount Hermon are characterised by low ambient temperatures (T_a_s) and snow during the winter [27]. In this area of the eastern Mediterranean, many species of plants and animals originating from different zoogeographical origins (e.g. Ethiopian and Palaearctic) coexist and reach the limits of their geographical ranges [28]. For example, the common spiny mouse *Acomys cahirinus*, of Ethiopian origin, which has the northern-most limits of its distribution in southern Turkey [29], coexists with the broad-toothed field mouse *Apodemus mystacinus*, of Palaearctic origin, which has the southern-most limits of its distribution in the Judean hills [30]. The fact that such species are at the edges of their geographical distribution ranges is possibly indicative of limits to their ecophysiological abilities to cope with the environmental challenges encountered in these habitats [12]. Therefore, this offers a valuable natural opportunity with which to explore physiological responses to environmental challenges.

Populations of *A. mystacinus* are found in the Mediterranean ecosystem in Israel as on the coastal slopes of Mount Carmel (Mediterranean mice), with mean T_a_s in January and August of 12 and 26°C, respectively, and a mean annual precipitation of 550 mm. Populations of this species also exist on the slopes of Mount Hermon on the eastern side of the Great African Rift Valley at altitudes of 1650 m (sub-Alpine mice), a sub-alpine ecosystem with mean T_a_s in January and August of 6 and 22°C, respectively, and a mean annual precipitation of over 900 mm [31]. These two populations appear to have been genetically separated for some time, having originated from different invasions [32], [33]. In the Carmel, severe shortages of water at the end of long hot and dry summers have been suggested to present the most problematic environmental challenge for this population [34], [35]. During this season, the vegetation is expected to have an increased particle (or osmotic) concentration due to the high evaporation of water as a result of increased solar radiation [36]. Indeed, increased salinity in the water source or food can effectively induce dehydration responses to both VO_2_ and T_b_ [16], [37]. By comparison, cold winters and snow are presumed to present the most challenging conditions for active (non-hibernating) mice inhabiting the sub-alpine ecosystem [34]. Bearing in mind the differences between the two ecosystems, Mediterranean mice may face greater challenges with respect to osmotic load whereas sub-Alpine mice may face greater challenges with respect to low T_a_s. Results of previous studies have revealed that acclimation to short photoperiod is an environmental cue for winter acclimatization of the thermoregulatory system whilst acclimation to long photoperiod simulates heat acclimation or summer acclimatization [4], [14], [38]–[41] We therefore propose the following hypothesis: “If Mount Carmel is a less thermally but more xerically challenging environment than Mount Hermon, the latter sub-Alpine population is predicted to be more responsive to changes in photoperiod such as short day conditions (simulating winter) whereas the former Mediterranean population should be more responsive to osmotic load (simulating aridity)”. Consequently we aimed to examine whether physiological responses to photoperiod and osmotic load would reflect differences in the abilities of mice from these two populations to cope with the seasonal challenges faced in their natural environments. Although it is not possible to establish the effects of an independent variable, such as salinity or photoperiod, by the study of only two species or populations that differ in this trait [42], we present the results of VO_2_ and T_b_ daily rhythms, variation in Heterothermy index (HI), a species-independent index of T_b_ [43], as well as energy intake for two populations of *A. mystacinus* as an indication of the role that climatic differences might play in shaping physiological diversity.

## Methods

### Ethics Statement

Permission was granted from the Nature and Parks Authority of Israel to collect mice from both field sites (below). The protocol was approved by the committee on the ethics of animal experiments of the University of Haifa (permit number 016 2002). The study was performed in accordance with the recommendations in the Guide for the Care and Use of Laboratory Animals of the National Institutes of Health.

### Animals

Studied individuals of *A. mystacinus* were captured from a site on the slopes of Mount Hermon representing a sub-alpine ecosystem (35°00′ E 33°00′ N; 1650 m elevation) in December 2002 (‘sub-Alpine’mice) and in June 2003 and from Har Horshan region on the Carmel ridge representing a Mediterranean ecosystem (32°43′ E 34°58′ N; 100 m elevation) in March 2003 (‘Mediterranean’ mice). Young or juvenile animals and females that were obviously pregnant or lactating were released. Mice were taken to the laboratory at the University of Haifa Oranim campus, Kiryat Tivon where they were maintained individually in cages (35×25×15 cm) and provided with sawdust and tissue paper as bedding material. Mice were kept in the laboratory for at least three weeks to establish whether any of the females were pregnant. No females were pregnant. They were acclimated to laboratory conditions after capture from the wild for at least two months (short-day; ‘SD’ - 8L: 16D, lights on between 08∶00–16∶00 h) before any measurements were taken. They were offered dried rodent chow (21% crude protein, 4% crude fat, 4% cellulose, 13% moisture, 7% ash, 17.4 kJ/g gross energy; Koffolk, Israel) *ad libitum* and agar gel (20 g of agar gel dissolved in 1000 ml of water) as a source of moisture. Mice were then subjected to various measurements of different physiological variables (digestibility, VO_2_, and T_b_ measurements, sections 2.3, 2.4 and 2.5) at three different salinity levels. The same mice were used for the different experiments which were always conducted in the same sequence, i.e. digestibility, VO_2_ and then T_b_ measurements. They were then acclimated to a long photoperiod (long day; ‘LD’ - (16L: 8D, lights were on between 06∶00–22∶00 h) for two months and all measurements were repeated. These photoperiod regimes simulated thermogenic acclimation that would have been a result of seasonal acclimatization in the wild, as photoperiod is the primary cue for seasonal acclimatisation [4], [35], [41]. In this case, T_a_ was kept constant at 25±0.1°C throughout the entire experimental period. The intensity of light during the photophase was 450 lux, which was provided by white fluorescent lighting with a dominant wave length of about 470 nm. A dim red light in the corner of the room was on continuously (25 lux) which allowed experimenters to work during the ‘dark’ periods [44], [45]. The current study is a continuation of previous work by the authors on measurements of the daily rhythms of T_b_ and VO_2_ in *A. mystacinus* [6], of measurements from Mount Hermon [34] and Mount Carmel [35] populations, and of osmoregulatory variation in Mediterranean and sub-Alpine mice [46]. There is no data overlap with the present study.

### Osmotic Loading

To simulate changes of osmotic load experienced in the wild across the seasons, animals were exposed to different levels of osmotic load by altering the level of salt (NaCl) dissolved into the agar that was provided as their water source [12], [46]. We dissolved NaCl in the agar at concentrations of 0.9%, 1.4% and 1.8%; the lowest salinity (0.9%) representing the osmotic loading that they would experience in the wild [36]. Several physiological variables were then measured for each of the six different treatment conditions (i.e. for the two photoperiods and the three salinity levels) for mice from both ecosystems. Acclimation to each increase in salinity, at a given photoperiod regime, lasted for two weeks (which is sufficient time for acclimation of the thermoregulatory system [47]–[50]).

### Energy Intake

Mice were transferred to cages with paper towelling (4 sheets, approximately 40×30 cm per sheet), with no sawdust at the beginning of a 7-day trial period. They were offered an amount of food (c. 80 g ±0.01 g) dried to constant weight. One week later, the remaining food and the faeces produced were removed from each cage and dried to constant weight in an oven (Memmert, Germany; 60°C). Subsequently, for each mouse, at each level of salinity and photoperiod regime, the dry food and faeces remains were separated by hand, weighed (Sartorius, Germany; ±0.01 g), homogenised, and the faecal calorific value (FE, kJ/g) determined by adiabatic bomb calorimetry (Semi-micro Calorimeter 1425. Parr, USA). We assume that bacterial breakdown of faeces was negligible since conditions were dry and even under ideal conditions faecal breakdown for this time period is typically less than 10% [51]. We obtained measures of food intake and faecal output at each level of salinity and photoperiod regime. We determined dry matter intake (DMI, g) as the mean dry food consumed per day and faecal output (FO, g) as the mean dry faeces produced per day. Both variables were calculated per gram body mass (W_b_). Apparent digestible energy intake (ADEI, kJ/day) and digestible efficiency (DE) was then calculated [52] as:
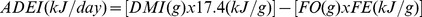
(1)

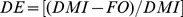
(2)


### Daily Rhythms of Oxygen Consumption (VO_2_)

Oxygen consumption (VO_2_) daily rhythms were monitored using a 6-chamber switching device in an open circuit system [53], [54]. Five metabolic chambers (5020 ml volume for each) were used for measuring VO_2_ with the sixth used to obtain a baseline. Each chamber was sampled in turn and concentrations of O_2_ in the dried efflux air were monitored in 100 s time bins with flush-out times of 100 s [55], [56]) using a Servomex Xentra 4100 automated oxygen analyser linked to a computer (Logal hardware and software for VO_2_ analysis, MODCON systems, Wonderware InTouch 7,1,0,0; Tuchenhagen, Ireland, Ltd.). Oxygen consumption readings were taken during the stable period after the 100 s flush-out which provided measurements of VO_2_ for each mouse approximately every 20 minutes. We verified that there were no air leaks in the system before each measurement. Air was dried before entry into the metabolic chambers by passing it through a freeze-dryer at −70°C, and again before entering the analyser. The flow rate through each chamber was set to 500 ml/min (mass-flow meter, Modcon systems, Israel) which was calibrated with a bubble flowmeter [57]. The oxygen analyser was calibrated to an upper value (20·95% O_2_, atmospheric air) prior to each measurement and to a lower value (0·0% O_2_, N_2_ gas) every 2 weeks. The thermocouples within the metabolic chamber were calibrated to 0.0°C prior to each measurement by placing them in ice water. Mice were placed in the chambers at midday for 72 h with food and agar gel provided *ad libitum* (c. 50 g of rodent chow and 20 g of fresh agar was placed in the chamber with each animal). Chambers were placed inside an incubator (Labline), which was set at 25°C and had an internal lighting regime (including a dim red light) that was consistent with laboratory acclimation conditions. Four sheets of paper towelling (40×30 cm per sheet) were provided as bedding material. We analysed the final 48 h of VO_2_ data and not the first 24 h to allow mice to become accustomed to chamber conditions.

### Daily Rhythms of Body Temperature (T_b_)

Rectal T_b_s were measured for a total of 36 h using a copper-constantan thermocouple connected to a TH-65 Wescor thermometer. To minimise the disturbance to each animal, mice were measured in two batches with only half of the animals measured every four hours thus each individual animal was measured only once every eight hours [58]. For each measurement, the thermocouple was inserted 2.5 cm into the rectum of a mouse for no more than 30 seconds. We did not implant data logging devices or transmitters because we had ethical concerns working with such small (20–30 g) animals [59], [60].

### Statistics

General linear models were used to determine differences between groups in DMI and ADEI and a Generalized Linear Model with a logit link function was used to assess differences in DE. Body mass and salinity entered as covariates while habitat and photoperiod were entered as factors [61]. Cosinor analysis was used to determine the VO_2_ and the T_b_ daily rhythms of measured individuals with the period set to 24 h. The mean (mesor) values, amplitude and the acrophase of the VO_2_ and T_b_ daily rhythms were calculated for each individual [62], [63]. While cosine curves adequately fitted the VO_2_ data, they were less adept at fitting the T_b_ data. Therefore, Repeated measures ANOVA was used to examine the variation in cosinor parameters (mesor, amplitude, acrophase) of VO_2_ with habitat and photoperiod entered as factors. Restricted maximum likelihood was used to estimate the model parameters. By comparison, general additive models were used to examine variation in T_b_ data [64]–[66]. Finally, variation in HI was reported. Heterothermy index was calculated as the square root of the sum of the squares of the individual differences between the modal T_b_ and each individual T_b_ [43] for each animal, under each condition. Relationships between photoperiod, habitat and salinity with HI were investigated using general linear models.

## Results

### Energy Intake

**Figure 1:**
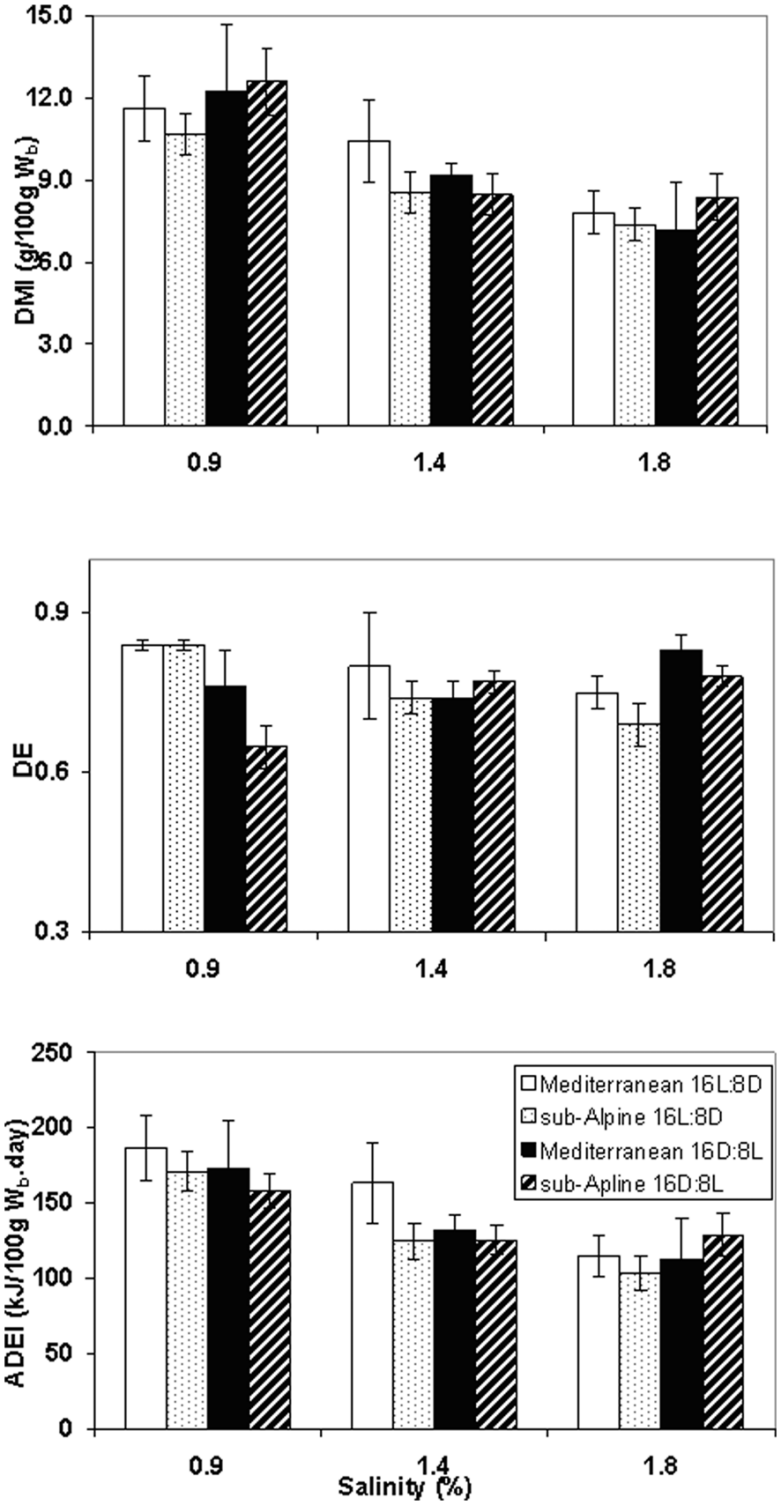
Digestibility Experiments. (**A**) Dry matter intake (**DMI**: g/100 g W_b_.day); (**B**) Digestible efficiency (**DE**) and (**C**) apparent digestible energy intake (**ADEI**: kJ/100 g W_b_.day) of *A. mystacinus* at salinity regimes of 0.9, 1.4 and 1.8%. Open and dotted bars denote long-day (16L:8D) Mediterranean and sub-Alpine mice; dark bars and hashed bars denote short-day (16D:8L) Mediterranean and sub-Alpine mice, respectively. “/100 g W_b_.day” denotes per 100 g body mass per day. Error bars denote standard errors.

Mean body mass of all animals was 32.1±5.67 g. There were no effects of habitat, photoperiod or salinity on body mass (F_1,105_ = 0.51, p = 0.475; F_1,105_ = 0.13, p = 0.717 and F_1,105_ = 0.18, p = 0.677 respectively, Table 1). There were consistent differences in body mass between individuals, with large individuals remaining large. Dry matter intake decreased with increasing salinity in mice from both populations and under the two photoperiod regimes (F_1,104_ = 34.68, p<0.001) (Fig. 1A). Mediterranean mice had significantly greater DE values than sub-Alpine mice (χ^2^ = 5.33, p = 0.021) (Fig. 1B). A significant interaction was noted between photoperiod and salinity (χ^2^ = 22.33, P<0.001); DE values were greater for mice exposed to long days at low levels of salinity but greater for mice exposed to short days at higher levels of salinity. For ADEI the overall effect of increasing salinity resulted in a decrease of energy intake in both populations. However, there was a significant interaction between photoperiod and salinity with ADEI higher in LD- than in SD-acclimated mice at low levels of salinity but not at higher levels (F_1,103_ = 4.42, P = 0.034) (Fig. 1C).

**Table 1:** Daily rhythms of oxygen consumption and body temperature.

	CARMEL								HERMON						
	SD				LD				SD				LD		
**Salinity**	**0.9**	**1.4**	**1.8**		**0.9**	**1.4**	**1.8**		**0.9**	**1.4**	**1.8**		**0.9**	**1.4**	**1.8**
n	7	7	9		8	8	8		13	9	12		11	6	11
body mass (g)	30.37	32.73	31.28		32.40	30.10	31.05		30.21	33.01	33.15		32.91	34.08	32.93
se	4.02	2.14	2.19		2.31	1.00	1.08		1.78	2.13	2.43		1.39	2.71	1.60
**VO_2_ daily rhythms**															
n	5	6	5		7	7	7		7	7	7		8	8	8
Mesor (ml O_2_/g.h)	2.11	3.17	4.03		3.34	2.94	3.33		1.91	2.87	2.14		2.36	2.52	2.21
Amplitude (ml O_2_/g.h)	0.660	0.695	0.463		0.876	0.537	0.722		0.322	0.517	0.517		0.634	0.418	0.574
Acrophase	2∶58	2∶01	2∶58		1∶24	1∶48	1∶55		23∶24	2∶31	0∶02		1∶47	0∶32	0∶37
% rhythm	63.90	63.92	55.56		43.30	42.79	44.69		54.97	50.79	65.20		50.24	43.50	37.25
p value	0.05	0.02	0.10		0.04	0.03	0.02		0.05	0.03	0.15		0.05	0.002	0.02
**T_b_ daily rhythms**															
n	8	10	11		9	8	8		13	11	12		11	11	11
Mesor (°C)	37.03	37.13	0.35		37.31	38.15	38.02		37.71	37.26	37.22		37.80	38.18	38.45
Amplitude (°C)	0.651	0.130	36.610		0.244	0.282	0.812		0.214	0.558	0.182		0.107	0.093	0.165
Acrophase	2∶41	11∶36	19∶41		14∶22	22∶48	0∶19		7∶44	10∶18	8∶33		0∶44	15∶08	23∶20
% rhythm	61.81	2.41	58.46		15.80	32.64	84.93		19.24	23.36	6.49		4.52	6.30	31.17
p value	0.01	0.91	0.03		0.60	0.31	0.003		0.36	0.35	0.76		0.87	0.83	0.33

Mean and standard errors (se) of body mass (g) as well as mesor, amplitude and acrophase values of oxygen consumption (VO_2,_ mlO_2_/g.h) and body temperature (T_b_, °C) daily rhythms of *A. mystacinus* from Mount Carmel (‘Carmel’) and Mount Hermon (‘Hermon’) acclimated to short day (SD, 16D: 8L) and long day (LD, 16L:8D) photoperiods and to salinity regimes of 0.9, 1.4 and 1.8%. Percent rhythm and probability (p values) of the goodness of fit of the cosine curve [63] are shown.

### Daily Rhythms of Oxygen Consumption (VO_2_)

**Figure 2:**
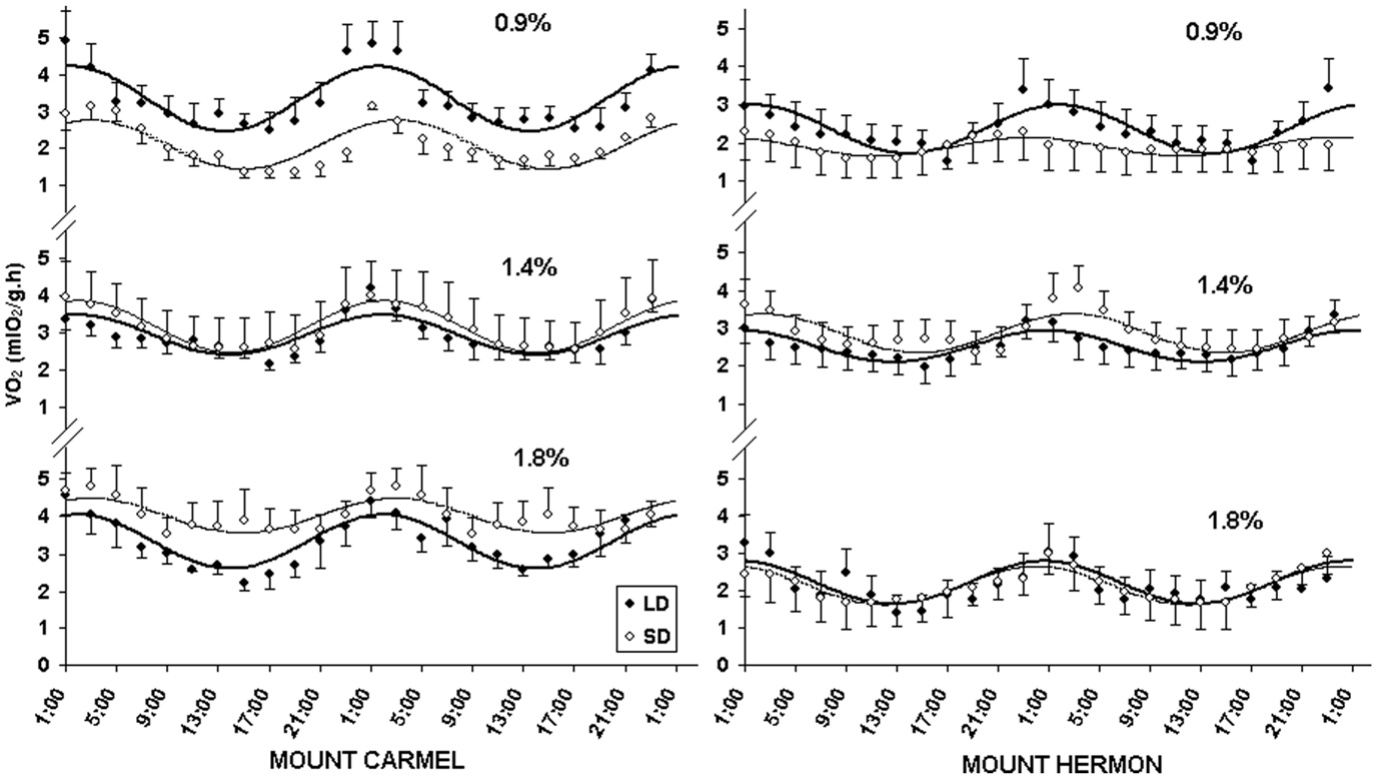
Oxygen consumption daily rhythms. Oxygen consumption (**VO_2_:** mlO_2_/g.h) daily rhythms of short day (**SD:** open circles, continuous line) and long day (**LD:** closed circles, bold line) acclimated *A. mystacinus* at salinity regimes of 0.9, 1.4 and 1.8% from Mount Carmel and Mount Hermon. Curves denote best-fit cosine functions [54]. Error bars denote standard errors.

There were differences in VO_2_ between populations under the different challenges. Mesor values were significantly higher in Mediterranean- than sub-Alpine mice (F_1,6_ = 8.02, p = 0.050) (Table 1, Fig. 2). Compared to mice exposed to long days, those exposed to short days maintained higher VO_2_ values when given water with high salinity than they did when given water with low salinity (F_1,6_ = 32.82, p<0.001). By comparison, amplitude values were significantly greater in Mediterranean than sub-Alpine mice (F_1,6_ = 6.15, p = 0.048), however, there were no effects of photoperiod or salinity (F_1,6_ = 0.19, p = 0.681; F_1,6_ = 0.08, p = 0.932, respectively). Finally, acrophase also differed significantly between populations (F_1,6_ = 8.89, p = 0.025); Mediterranean-mice exhibited a VO_2_ peak at approximately 02∶30 h while sub-Alpine-mice showed a peak at approximately 00∶45 h (Fig. 2).

### Daily Rhythms of Body Temperature (T_b_)

**Figure 3:**
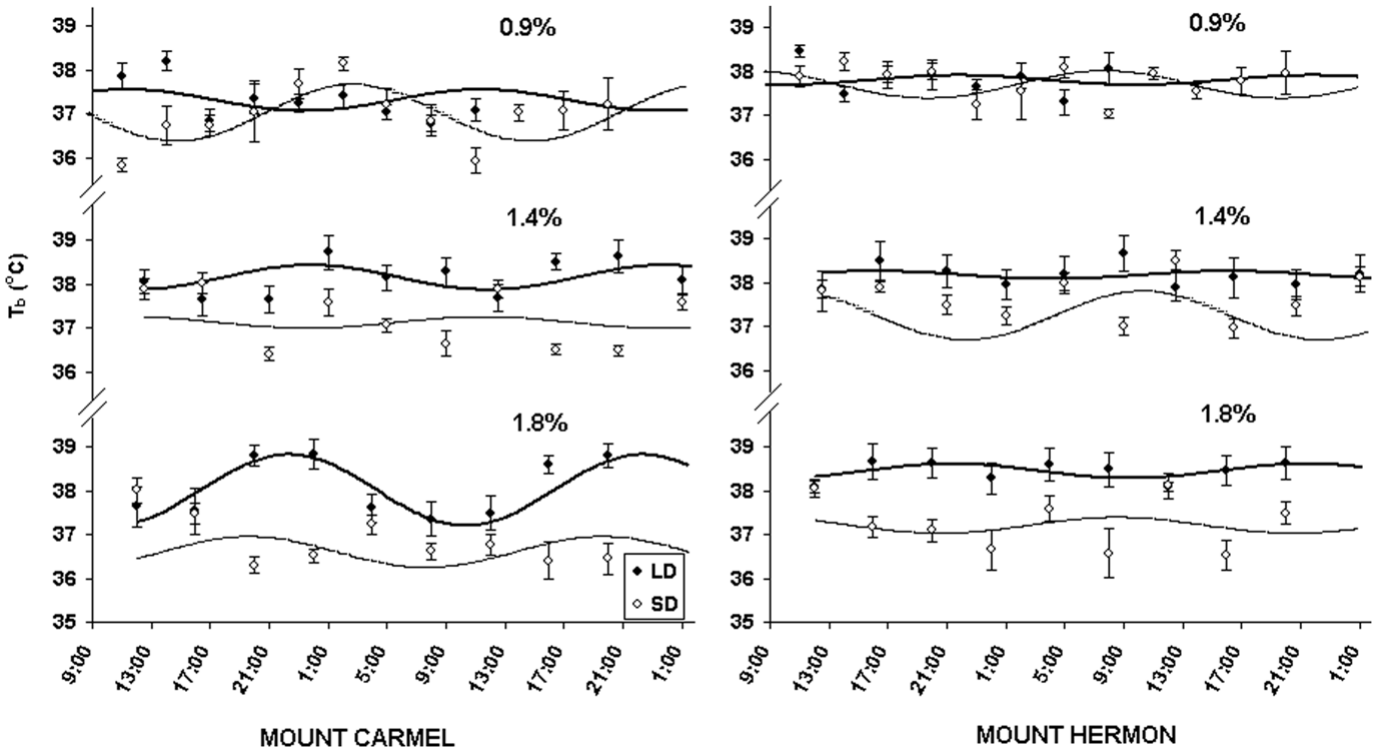
Body temperature daily rhythms. Body temperature (**T_b_:** °C) daily rhythms of short day (**SD:** open circles, continuous line) and long day (**LD:** closed circles, bold line) acclimated *A. mystacinus* at salinity regimes of 0.9, 1.4 and 1.8% from Mount Carmel and Mount Hermon. Curves denote best-fit cosine functions [54]. Error bars denote standard errors.

Significant differences between populations under the different photoperiod regimes and different salinity levels were noted in the variables describing the T_b_ daily rhythms. T_b_ values were significantly higher in sub-Alpine- than Mediterranean mice (F_1,7_ = 19.93, p<0.001) (Fig. 3). Compared to mice exposed to short days, those exposed to long days maintained warmer bodies when given water with high salinity than they did when given water with low salinity (F_1,7_ = 26.90, p<0.001). Under the different salinity conditions acrophase responded significantly to photoperiod in mice from both populations (F_1,8_ = 8.02, p = 0.022). Mean HI value across all individuals was 0.453±0.463°C. Compared with mice exposed to long days, those exposed to short days maintained higher HI values when given water with low salinity (0.9% and 1.4%) than they did when given water with a high salinity (1.8%) (F_1,116_ = 8.31, p = 0.005).

### Combined Energy Intake and Energy Expenditure

**Figure 4:**
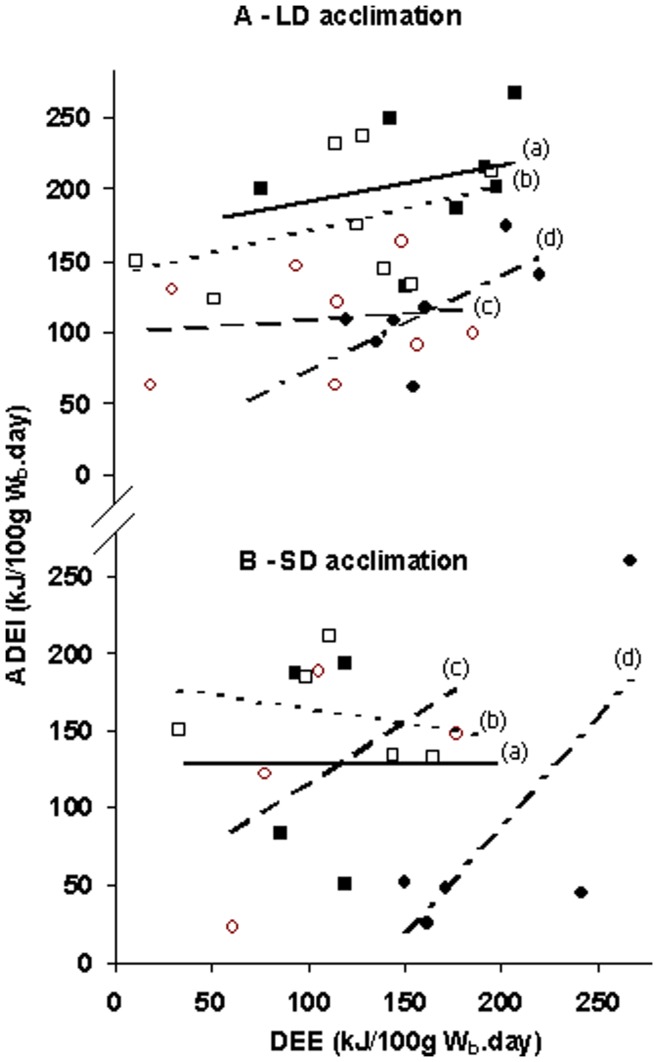
Energy intake and energy expenditure. Energy intake (Apparent digestible energy intake, **ADEI**, kJ/100 g W_b_.day) measured by food intake and faecal output against energy expenditure (**DEE**, kJ/100 g W_b_.day) measured by oxygen consumption for Mount Carmel (**MC**) and Mount Hermon (**MH**) *A. mystacinus* acclimated to (**A**) long day (**LD**; 16L:8D) and (**B**) short day (**SD**; 16D: 8L) photoperiod regimes. 0.9% salinity MC mice are denoted by solid squares and a solid line (‘a’); 0.9% salinity MH mice are denoted by open squares and a dotted line (‘b’); 1.8% salinity MH mice are denoted by open circles and a hashed line (‘c’) and 1.8% salinity MC mice are denoted by solid circles and a dot-hashed line (‘d’). “/100 g W_b_.day” denotes per 100 g body mass per day.

Average daily energy intake (ADEI) calculated from food intake and average daily energy expenditure (DEE) calculated from VO_2_ data were compared. Whilst there was no direct effect of habitat under LD-acclimation, there was a significant effect of salinity with mice from lower salinity regimes having a greater ADEI than those from higher salinity regimes (F_2,39_ = 20.34, p<0.001) (Fig. 4A). By comparison, under SD-acclimation, the effect of salinity was weaker but mice from lower salinities continued to have greater energy intakes than those under higher salinity regimes (F_2,19_ = 4.49, p = 0.025) (Fig. 4B). The interaction between salinity and habitat failed to reach significance (F_2,19_ = 2.90, p = 0.079). This effect was caused primarily by the different responses of mice on 1.8% salinity compared with mice on lower salinity regimes. Post-hoc analyses revealed that, at 1.8% salinity and SD conditions, there was a significant interaction between habitat and photoperiod (F_1,18_ = 5.56, p = 0.030) with Mediterranean mice having a higher energy expenditure relative to energy intake than sub-Alpine mice.

## Discussion

Variation in physiological capabilities between populations inhabiting different environments has been suggested to provide an insight into the dynamics of evolutionary changes [1], [67]. Across different zoogeographical zones, there is a large corresponding variation in small mammal energy metabolism [68], [69]. Physiological variations within a population may allow adaptation to environments differing in temperature, aridity and productivity [70], [71]. Comparing differences in the daily rhythms and photoperiod-induced changes of physiological characteristics can add another important dimension, namely responses to a temporal environment. Various studies have shown that changes in osmotic loading can affect the VO_2_ and T_b_ of small mammal populations from different habitats or ecosystems in different ways [12], [16], [35] and that this variation might indicate physiological plasticity or adaptation to local conditions [6], [46]. In the current study, we used two environmental signals - photoperiod and osmotic load - that affect energy metabolism and body temperature regulation in different ways. Our setup can therefore be considered: (1) when both signals convey the same season (e.g. either ‘summer’ - LD and high osmotic load or ‘winter’ - SD and no osmotic load), and (2) where signals convey conflicting information (e.g. SD and high osmotic load or LD and no osmotic load) (Table 2). The interaction between these effects is of particular importance in elucidating site-dependant differences in the sensitivity to these signals, which may indicate population level differences in physiological characteristics.

**Table 2:** Expected ambient conditions for each treatment.

Photoperiod	Habitat	Expected	Expected	Expected	Salinity	Conflicting
		T_a_	aridity	osmolarity	regime	signal
**Short day**	Mediterranean	Cool	mesic/wet	low	0.9	No
				low	1.4	
				low	1.8	Yes*
	sub-Alpine	Cold	freezing	low	0.9	No
				low	1.4	
				low	1.8	Yes*
**Long day**	Mediterranean	Hot	arid	high	0.9	Yes**
				high	1.4	
				high	1.8	No
	sub-Alpine	Hot	arid	high	0.9	Yes**
				high	1.4	
				high	1.8	No

Expected conditions of ambient temperature (T_a_), aridity, and osmolarity of the food source for Mount Carmel (Mediterranean) and Mount Hermon (sub-Alpine) mice exposed to short day (winter) and long day (summer) conditions. The laboratory conditions of short day (8L:16D) and long day (16L:8D) and salinity regimes of the drinking water source (0.9%, 1.4% and 1.8%) present mice with environmental signals which can either represent what occurs in the wild, in which case the environmental signals are not conflicting (No); or they can represent different conditions to what the mice experience in the wild, in which case the environmental signals are conflicting (Yes). * indicates a potentially stressful situation as mice do not expect a high salinity load; ** indicates a potentially stressful situation as mice do not expect a low salinity load.

### Energy Intake

The general physiological response to increased osmotic load is a reduction in food intake [72] which reduces stress on the kidneys by decreasing urea secretion. In the current study, there were no effects of salinity on body mass. However, we did note that *A. mystacinus* could not maintain their body mass when offered a salinity loading of 2.5% (M. Scantlebury and A. Haim unpublished data). Hence, one can conclude that while 1.8% osmotic loading did have a physiological effect on the mice, the maximum osmotic load chosen may not have been reached. For both populations, DMI and ADEI decreased with increasing salinity, confirming the expected response. However, there was also an interaction between photoperiod and salinity for DE and ADEI. LD-mice had greater DE values at the low salinity whereas SD-mice had greater DE values at the highest salinity. In general, animals living in less productive environments have an increased digestive efficiency, compared with those from more productive environments [22]. There were differences in the rates and digestive efficiency between populations of common spiny mice acclimatised to different seasons and acclimated to different photoperiod regimes [73], [74]. The fact that SD-mice in the current study were able to increase DE at a higher salinity load suggests that they were able to conserve energy by utilising the food source more efficiently and conserve water by decreasing faecal output [74]. However, the fact that LD-mice decreased their DE with increasing salinity suggests that they may have reduced water loss in other avenues, for example by producing dryer faeces, by dissipating more heat through non-evaporative routes or by increasing the efficiency of their kidneys [46]. Hence, the LD signal can be presumed to ‘prepare’ mice for the dry season, which is characterised by water limitations and osmotic load [41]. Indeed, under the two higher salinities, LD-mice had significantly higher mesor values of T_b_ than SD-mice (Fig. 3).

### Daily Energy Expenditure (DEE)

In a previous study in which the VO_2_ daily rhythms of Mediterranean-mice were measured under a photoperiod of 12L:12D at their lower critical temperature and fresh carrots provided as a water source [6], the mesor values obtained (2.89 mlO_2_/g.h) were similar to those of SD and LD-mice when they were kept on 1.4% NaCl. Of note is that salinity had a large effect on SD-mice (Fig. 2). In particular, increasing salinity resulted in increased VO_2_ values in SD Mediterranean-mice whereas no such change was observed in LD-mice. This may be because SD is the environmental signal for water availability in Mount Carmel, which results in an expected reduction in osmotic load [46]. However, when photoperiod and salinity signals were presented in opposing directions (i.e. SD and high salinity) the response of mice was an increase in energy expenditure. It can be presumed that this shows a potential energetic cost of osmoregulation for mice not ‘accustomed’ to dealing with these ‘confusing’ conditions. In the case of sub-Alpine mice, a smaller increase in VO_2_ was noted when salinity was increased under SD-acclimation compared with Mediterranean mice. One possibility why sub-Alpine mice had a reduced response to photoperiod might be that temperature is a more important environmental cue for them than salinity. Results of a previous study on *A. mystacinus* showed that sub-Alpine-mice exhibited a higher resistance to cold and significantly higher VO_2_ values upon exposure to low ambient temperature compared with Mediterranean-mice [34]. This would make sense as the environment in Mount Hermon is manifest as greater seasonal variability in T_a_ compared with that of Mount Carmel. The lack of response to photoperiod observed in sub-Alpine-mice could therefore be due in part to lack of exposure to low T_a_s (at or below 6°C).

Interestingly, Mediterranean-mice had greater amplitudes of variation than sub-Alpine-mice (Fig. 2). One possibility might be that Mediterranean mice were attempting to conserve water and energy by reducing the T_b_-to-T_a_ gradient [60] suggesting that Mediterranean mice might be more suited to live in conditions with high osmotic load. Finally, there were differences in acrophase between the two populations with sub-Alpine mice peaking in VO_2_ earlier in the dark phase than Mediterranean mice, indicating differences in activity patterns (Fig. 2). These differences could emerge for several reasons, both biotic and abiotic. For example, *A. mystacinus* on Mount Carmel coexist with the competitively superior common spiny mouse *Acomys cahirinus* that has been suggested to lead to temporal exclusion [75]. It is also possible that differences in acrophase occur because there are differences in T_a_ values between the habitats - i.e. as nocturnal animals; sub-Alpine-mice might be active earlier than Mediterranean mice, before the night becomes too cold.

### Balance of Energy Intake and Energy Expenditure

There were differences in energy flux between the different groups of mice. This can be seen as differences in the gradients of the graphs describing the relationships between ADEI and DEE under LD acclimation (Fig. 4). Under SD and a high salinity load, the gradient [i.e. delta (X)/delta (Y)] between ADEI and DEE increased and mice expended more energy relative to food intake, whereas the opposite was true for a low salinity load. If it is assumed that activity was independent of salinity, then one possibility is that the kidneys utilised energy to process the high levels of salt intake. This was more notable for SD-mice, which as previously suggested, may not be ‘expecting’ osmotic load under this photoperiod.

### Body Temperature Daily Rhythms

Body temperature daily rhythms are an outcome of two different other rhythms, namely heat production and heat dissipation and are suggested to be related to feeding and activity patterns [6], [76], [77]. Short photoperiod acclimated fat jirds (*Meriones crassus*) had longer periods of maximal T_b_ compared with long photoperiod acclimated individuals under the same T_a_, which was interpreted as an adaptation enabling jirds to forage for longer periods during the winter [14]. Similarly, Rubal et al. [6] suggested that higher T_b_ values for a longer period within the 24 h cycle indicated adaptation to a cooler environment in *A. mystacinus*. The results of the current study can be compared with those obtained earlier on *A. mystacinus* from Mount Carmel and Mount Hermon populations in which mice were not challenged with photoperiod manipulations [6] or salinity [34], [35]. Values obtained for Mediterranean mice for both photoperiod regimes under a salinity of 0.9% were significantly lower than those obtained previously (38.44±0.06°C) [6] (p<0.001 for both LD and SD acclimation). Sub-Alpine mice in the current study also had lower values than sub-Alpine mice acclimated to 12L:12D at the same ambient temperature [34]. However, for both populations, the values of LD-acclimated mice at 1.4% and 1.8% were similar to those that appear in literature [6], [34]. The reasons why salinity may be linked to T_b_ may emerge from the fact that LD conditions co-occur with increasing salinity (osmolality or particulate matter) in food items and higher T_a_’s. Hence, by providing opposing signals to mice (e.g. high salinity under SD conditions) one ‘confuses’ their thermoregulatory system. The higher T_b_ values noted in LD-mice under high salinity of both populations may confer an advantage for heat dissipation under restricted water conditions. However, SD-mice did not increase their T_b_ values with increasing salinity. This can be understood in terms of their inability to raise T_b_ in order to dissipate heat at a time of the year (i.e. short photoperiod conditions representing winter) when water deprivation and osmotic load do not co-occur. Interestingly, at 1.8% salinity, LD sub-Alpine mice did not show a T_b_ daily rhythm pattern whilst LD Mediterranean mice had a clear pattern. This is consistent with previous studies which show that both seasonal acclimatization (July) and acclimation to LD resulted in arrhythmic T_b_ daily rhythms in Mediterranean mice with no water stress [35]. A possible interpretation of the fact that a high salinity signal resulted in a clear daily rhythm pattern only for Mediterranean mice is that salinity is likely to be a more important cue for challenging Mediterranean mice; whereas, as previously mentioned, other cues such as low temperature could be more important for sub-Alpine mice. Finally, both photoperiod and salinity had a significant effect on HI. Observed values were greater in SD than LD-acclimated individuals at lower levels of salinity, but greater in LD than SD-acclimated individuals at the highest level of salinity. This is consistent with the notion that animals are able to increase T_b_ variation when they are experiencing realistic ‘winter’ or SD conditions (i.e. SD and low salinity) or realistic ‘summer’ or LD conditions (i.e. LD and high salinity) but are less able to effect T_b_ variation when the ambient conditions are conflicting (i.e. SD and high salinity or LD and low salinity).

### Conclusions

Daily rhythms of thermoregulatory variables of two populations of mice from different ecosystems separated by the Great African Rift Valley were assessed for their responses to photoperiod and osmotic load. Mediterranean mice were more responsive to osmotic load than sub-Alpine mice, with salinity acting as a clear signal for changes in T_b_ daily rhythms and an increase of VO_2_. This indicates that Mediterranean mice may be able to respond to and cope with variations in osmotic load whereas sub-Alpine mice may be more sensitive to and able to cope with variations in ambient temperature [34]. In addition, LD-mice behaved differently to SD-mice upon exposure to increased salinity, with the former appearing to adopt mechanisms to reduce energy expenditure and water loss. Under LD-acclimation, mice were able to cope better with osmotic load which they may have been ‘expecting’ as a stressor.

The links between physiological adaptation and species distribution have received particular interest in the light of climate change and the requirements for prediction of future biodiversity [68], [78], [79]. Bacigalupe et al. [80] suggest that it is important to consider how the thermal environment may shape inter-population differences in physiology over evolutionary time. The results of our study highlight how daily rhythms of energy expenditure and T_b_ may vary across populations of the same species as a result of differences in photoperiod and water stress. Whilst we are aware that phenotypic plasticity may account for many of the differences noted between the populations, intraspecific comparisons such as this can be seen as a first approach towards understanding whether differences in traits can be adaptive.
